# The Concave Face of Decorin Mediates Reversible Dimerization and Collagen Binding*

**DOI:** 10.1074/jbc.M113.504530

**Published:** 2013-10-29

**Authors:** Mehwaesh Islam, Jayesh Gor, Stephen J. Perkins, Yoshihiro Ishikawa, Hans Peter Bächinger, Erhard Hohenester

**Affiliations:** From the ‡ Department of Life Sciences, Imperial College London, London SW7 2AZ, United Kingdom,; § Department of Structural and Molecular Biology, University College London, London WC1E 6BT, United Kingdom,; ¶ Research Department, Shriners Hospital for Children, Portland, Oregon 97239, and; ‖ Department of Biochemistry and Molecular Biology, Oregon Health and Science University, Portland, Oregon 97239

**Keywords:** Collagen, Extracellular Matrix, Protein-Protein Interactions, Proteoglycan, Site-directed Mutagenesis

## Abstract

Decorin, the prototypical small leucine-rich proteoglycan, binds to collagen and thereby regulates collagen assembly into fibrils. The crystal structure of the decorin core protein revealed a tight dimer formed by the association of two monomers via their concave faces (Scott, P. G., McEwan, P. A., Dodd, C. M., Bergmann, E. M., Bishop, P. N., and Bella, J. (2004) *Proc. Natl. Acad. Sci. U.S.A.* 101, 15633–15638). Whether decorin binds collagen as a dimer has been controversial. Using analytical ultracentrifugation, we determined a dissociation constant of 1.37 ± 0.30 μm for the mouse decorin dimer. Dimerization could be abolished by engineering glycosylation sites into the dimer interface; other interface mutants remained dimeric. The monomeric mutants were as stable as wild-type decorin in thermal unfolding experiments. Mutations on the concave face of decorin abolished collagen binding regardless of whether the mutant proteins retained the ability to dimerize or not. We conclude that the concave face of decorin mediates collagen binding and that the dimer therefore must dissociate to bind collagen.

## Teaser

**Background:** In the crystal structure of decorin, the concave faces of two monomers interact to form a tight dimer.

**Results:** The decorin dimer in solution is in equilibrium with stable monomers, and mutations on the concave face abolish collagen binding.

**Conclusion:** Decorin binds collagen as a monomer.

**Significance:** These findings help resolve the controversy about the functional oligomeric state of decorin.

## Introduction

The small leucine-rich proteoglycans (SLRPs)^2^ comprise a diverse family of secreted glycoproteins that have in common a core protein consisting of multiple leucine-rich repeats (LRRs) flanked by cysteine-rich cap regions. One or several glycosaminoglycan chains are attached to the canonical SLRPs; other family members have acidic regions or are modified by tyrosine sulfation (1, 2). Decorin is the prototypical SLRP. It has a well characterized role in regulating collagen fibrillogenesis (2, 3) and additionally modulates the activity of various growth factors and receptor tyrosine kinases (4). Ultrastructural studies of tissue-derived collagen fibrils have revealed decorin binding sites within the gap region of the D-period (5–7). A unique decorin binding site near the C terminus of the triple helix has been identified using type I procollagen produced in cell culture (8). Decorin inhibits collagen fibrillogenesis *in vitro* (9) and has a profound effect on the ultrastructure of the resulting fibrils (10). Decorin-deficient mice are viable and grossly normal but have fragile skin due to abnormal collagen fibrils (11). Mice lacking decorin and the related SLRP biglycan have a much more severe skin phenotype (12) and a severely disrupted collagen fibril architecture in the cornea (13).

**FIGURE 1.**
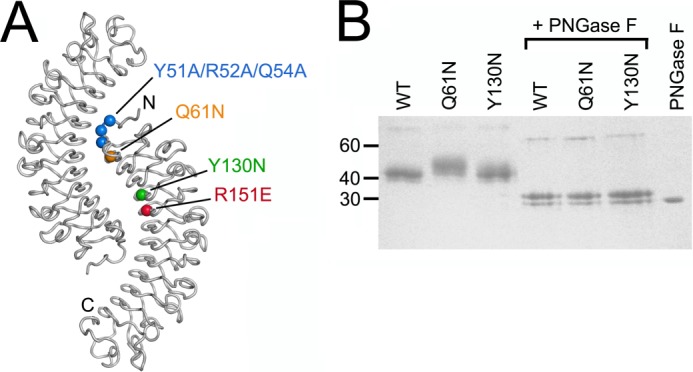
**Mouse decorin mutants.** *A*, location of mutations in mouse decorin mapped onto the crystal structure of the bovine decorin dimer (14). The dimer is viewed along its symmetry axis, and the N and C termini are labeled in one subunit. *B*, reducing SDS-PAGE of wild-type (WT) mouse decorin and the Q61N and Y130N mutants before and after digestion with peptide *N*-glycosidase F (*PNGase F*) (Coomassie Blue stain). The positions of selected molecular mass markers are indicated on the *left*.

The crystal structure of the decorin core protein revealed that the 12 LRRs form a curved solenoid; the concave face of the solenoid is a parallel β-sheet, and the convex back consists of irregular loops and single helical turns (14). In this crystal structure, two decorin monomers were found to interact through their concave faces, burying a large amount of decorin surface (see Fig. 1*A*). A strong tendency of decorin and other SLRPs to form dimers in solution was observed in several biophysical studies (15–17). Some even claimed that folded monomeric decorin cannot exist in solution (18), whereas others concluded that the crystallographic decorin dimer is an artifact (19). To complicate matters further, mutagenesis (20–22) and molecular modeling studies (23, 24) have implicated the concave face, which is largely buried in the decorin dimer, in collagen binding.

We felt that it was important to resolve the controversy about the oligomeric state of decorin and how it relates to collagen binding. Here, we show that decorin dimerization is relatively weak and reversible and that mutants that are stable monomers in solution can be obtained. Mutations on the concave face of decorin abolished collagen binding regardless of whether they disrupted the dimer or not. Thus, the same region of decorin mediates dimerization and collagen binding, and the decorin dimer must dissociate to bind collagen.

## EXPERIMENTAL PROCEDURES

### 

#### 

##### Expression Constructs

DNA coding for residues 45–354 of mouse decorin (UniProt P28654) was amplified by PCR from a full-length cDNA clone (OriGene) and inserted into a modified pCEP-Pu vector (25). After cleavage of the vector-encoded BM-40 signal peptide, vector-encoded APLA and AAAHHHHHH sequences are present at the N and C termini of the mature protein, respectively. The mutations were introduced by overlap extension PCR. All expression constructs were verified by sequencing.

##### Protein Production

The proteins were produced in human embryonic kidney HEK293 c18 cells (ATCC). The cells were grown at 37 °C with 5% CO_2_ in Dulbecco's modified Eagle's medium/F-12 (Invitrogen) containing 10% fetal bovine serum, 2 mm glutamine, 10 units/ml penicillin, 100 μg/ml streptomycin, and 250 μg/ml Geneticin. The cells were transfected with the pCEP-Pu expression plasmid using FuGENE (Roche Diagnostics) and selected with 1 μg/ml puromycin (Sigma). Confluent cells in a HYPERFlask (Corning) were washed twice with phosphate-buffered saline (PBS; Invitrogen) and incubated with serum-free medium for 3–4 weeks with weekly medium exchanges. The pooled and filtered conditioned medium was loaded onto a 5-ml HisTrap column (GE Healthcare) using an ÄKTA Purifier (GE Healthcare). The protein was eluted with 300 mm imidazole in PBS, concentrated using a Vivaspin centrifugal device (Sartorius), and further purified on a Superdex 200 16/60 size exclusion chromatography column (GE Healthcare) with Tris-buffered saline (TBS; 20 mm Tris, 150 mm NaCl, pH 7.5) as the running buffer. The fractions containing pure protein were pooled and concentrated to 2–3 mg/ml, and aliquots were flash frozen in liquid nitrogen. All experiments were performed with freshly thawed proteins. For analytical purposes, the *N*-linked glycan was removed by peptide *N*-glycosidase F treatment under denaturing conditions according to the manufacturer's protocol (New England Biolabs).

##### Analytical Size Exclusion Chromatography with Laser Light Scattering

Wild-type and mutant decorin samples at a concentration of 3 mg/ml (83 μm) were injected onto a Superdex 200 10/30 column (GE Healthcare) connected to a 1260 Infinity HPLC (Agilent Technologies). The running buffer was TBS, and the flow rate was 0.2 ml/min. Light scattering and refractive index changes were monitored using in-line Wyatt Mini Dawn and Optilab T-rEX detectors (Wyatt Technology Corp.). The data were analyzed with the Wyatt ASTRA V software using d*n*/d*c* values of 0.185 and 0.145 ml/g for the polypeptide and polysaccharide fractions of the glycoproteins, respectively. Each consensus site for *N*-linked glycosylation was assumed to add 2 kDa of molecular mass. The mass of the polypeptide fraction of the glycoproteins was determined by the three-detector method (26) using an extinction coefficient of 24,961 m^−1^ cm^−1^ for the decorin protein.

##### Analytical Ultracentrifugation

Sedimentation velocity experiments were performed at 20 °C using a Beckman XL-I analytical ultracentrifuge at a rotor speed of 40,000 rpm. The instrument was equipped with an eight-hole AnTi50 rotor with double sector cells with column heights of 12 mm. Sedimentation was monitored using absorbance (280 nm) and interference optics. Decorin proteins were dialyzed extensively against PBS and studied at concentrations ranging from 0.028 (0.77 μm) to 3.4 mg/ml (94 μm). The sedimentation boundaries were analyzed using direct boundary Lamm fits using the program SEDFIT (version 14.1) (27). A partial specific volume of 0.7289 ml/g was calculated from the amino acid and carbohydrate content. The buffer density and viscosity were taken to be 1.00543 g/ml and 1.02 centipoises, respectively, based on theoretical values provided by the program SEDNTERP. The continuous *c*(*s*) size distribution algorithm assumes that all species have the same frictional ratio *f*/*f*_0_ in each fit. The final SEDFIT analyses used a fixed resolution of 200 and optimized the *c*(*s*) fit by floating the meniscus, bottom of the cell, base line, and *f*/*f*_0_ ratio until the overall root mean square deviation and visual appearance of the fits were deemed satisfactory. The relative amounts of monomer and dimer were derived using the *c*(*s*) integration function. The dimer dissociation constants were obtained by fitting the ratio of monomer and dimer with Equation 1 using SigmaPlot 12.0 software (Systat Software Inc.).

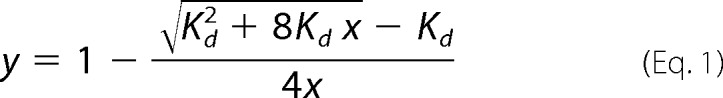
 where *y* is the dimer fraction, *x* is the total protein concentration, and *K_d_* is the dissociation constant. For a derivation of this equation, see Benfield *et al.* (28). HYDROPRO (29) was used to calculate *s*_20,*w*_^0^ values for the decorin monomer and dimer. The models were based on the crystal structure of dimeric bovine decorin core (14). Biantennary oligosaccharide chains, each consisting of a GlcNAc_2_Man_3_ core and two GlcNAc-Gal-NeuNAc antennae (30), were added at each of the four predicted glycosylation sites of mouse decorin, and the hydration shell was represented by an atomic element radius of 0.31 nm for all atoms (29).

##### Differential Scanning Calorimetry

The experiments were performed using a Calorimetry Systems Nano III calorimeter. Wild-type and mutant decorin samples at a concentration of 3 mg/ml (83 μm) were dialyzed extensively against PBS. 1-ml aliquots of sample and dialysis buffer were degassed for 15 min. Following an equilibration period of 10 min, initial scans from 5 to 20 °C were repeated until a stable base line was obtained. Scans were then performed from 5 to 65 °C at a heating rate of 1 °C/min.

##### Differential Scanning Fluorimetry

The experiments were performed using a Stratagene Mx3005p qPCR instrument essentially as described (31). 10-μl aliquots of wild-type and mutant decorin in PBS at a concentration of 0.362 mg/ml (10 μm) were mixed with 10 μl of SYPRO Orange solution (Invitrogen) diluted 1:250 and heated from 25 to 95 °C at a heating rate of 1 °C/min. The excitation wavelength was 492 nm, and fluorescence was monitored at 610 nm.

##### Collagen Fibrillogenesis Assay

A 1.05 mg/ml stock solution of mouse type I collagen (isolated from tendon, treated with pepsin, and precipitated using NaCl) in 50 mm acetic acid was neutralized at 4 °C by diluting it 33-fold with 150 mm sodium phosphate, 150 mm NaCl, pH 7.8 and immediately placed into a Shimadzu UV-2501PC spectrophotometer equipped with a water-jacketed cuvette holder maintained at 37 °C. The turbidity arising from collagen fibril formation was recorded as absorbance at 400 nm over 100 min. In the inhibition experiments, the decorin proteins were added at a concentration of 50 μg/ml (1.38 μm).

##### Solid-phase Binding Assay

A 1 mg/ml stock solution of rat tail type I collagen (Sigma) in 0.1 m acetic acid was diluted 1:40 with 50 mm Tris, 100 mm NaCl, pH 8.5 immediately prior to use. Nunc Maxisorp 96-well microtiter plates were coated overnight at 4 °C with 1.25 μg of collagen per well. The wells were washed once with PBS and blocked for 2 h at room temperature with 150 μl of 0.1 mg/ml bovine milk κ-casein (Sigma) in PBS containing 0.05% Tween 20 (incubation buffer). The cleared wells were incubated for 3 h with 50 μl of decorin proteins diluted in incubation buffer. After six washes with incubation buffer, the wells were incubated for 1 h with 50 μl of anti-His tag antibody conjugated to horseradish peroxidase (Miltenyi Biotec; 1:500 dilution in incubation buffer). After three washes with incubation buffer and three washes with PBS, the assay was developed using 75 μl of *o*-phenylenediamine dihydrochloride substrate (Sigma) per well for 20 min, and the reaction was stopped with 50 μl of 3 m H_2_SO_4_ per well. Absorbance was measured at 492 nm using a Tecan Sunrise microplate reader.

## RESULTS

### 

#### 

##### Mutational Disruption of the Decorin Dimer Interface

We created an expression construct for mouse decorin core protein (referred to as decorin from here on) that corresponds to the ordered residues in the crystal structure of bovine decorin core protein (14). This construct spans residues 45–354 (the numbering scheme includes the signal peptide) and contains a His_6_ tag at the C terminus. The C-terminal cap is not involved in the dimer interface of bovine decorin (Fig. 1*A*), and the His_6_ tag is therefore not expected to interfere with dimerization. We designed four mutations that might disrupt the dimer interface in mouse decorin (Fig. 1*A*). The Y51A/R52A/Q54A triple mutation in the N-terminal cap removes three side chains that are prominently involved in the interface (corresponding to Phe-27, Arg-28, and Gln-30 in the bovine decorin structure), the R151E mutation in LRR5 (Arg-127 in the bovine decorin structure) reverses a charge in the network of polar interactions that accounts for most of the interface, the Q61N mutation (Gln-37 in the bovine decorin structure) introduces a consensus site for *N*-linked glycosylation into LRR1, and the Y130N mutation (Tyr-106 in the bovine decorin structure) introduces a consensus site for *N*-linked glycosylation into LRR4. Analysis by the NetNGlyc server predicted glycosylation potentials of 0.60 and 0.72 for asparagines at positions 61 and 130, respectively, indicating a high probability that the engineered glycosylation sites would be modified.

Wild-type mouse decorin and all four mutants were obtained with good yields from episomally transfected HEK293 cells and purified to homogeneity (Fig. 1*B* and data not shown). The wild-type protein (calculated molecular mass, 36.2 kDa) ran as a single band of ∼43 kDa on reducing SDS-PAGE, consistent with the presence of four consensus sites for *N*-linked glycosylation in the mouse decorin sequence. The Q61N mutant ran as a broader band at higher molecular mass, demonstrating that the engineered glycosylation site at position 61 is modified by a glycan. In contrast, the electrophoretic mobility of the Y130N mutant resembled more closely that of the wild-type protein, indicating that the engineered glycosylation site at position 130 is either unmodified or that the attached glycan is not detectable by SDS-PAGE. Removal of the *N*-linked glycans by peptide *N*-glycosidase F digestion resulted in identical sharp bands at ∼30 kDa for the wild-type construct and the two mutants with engineered glycosylation sites (Fig. 1*B*).

##### Oligomeric States of Wild-type and Mutant Decorin

**FIGURE 2.**
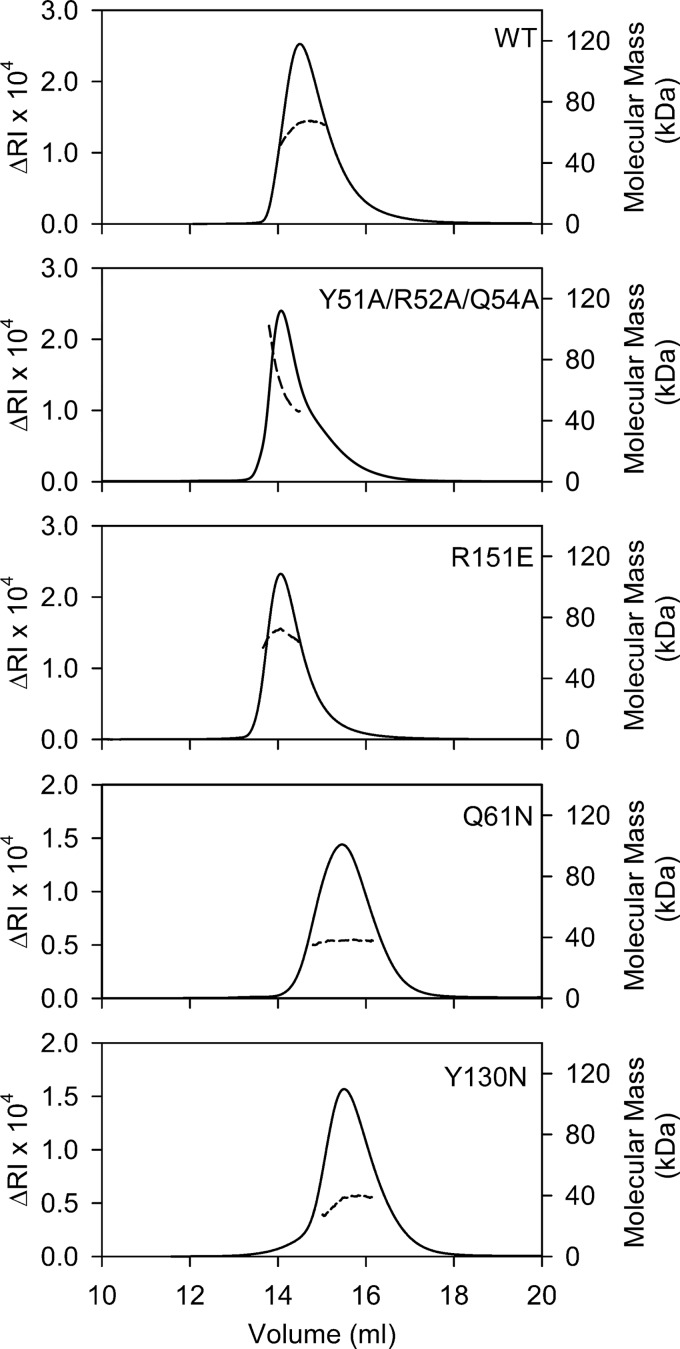
**SEC-MALS analysis of WT mouse decorin and the dimer interface mutants.** The proteins were injected onto a Superdex S200 column at a concentration of 3 mg/ml and run in TBS. The *solid lines* represent the refractive index (*RI*) detector signal (*left y axis*), and the *dashed lines* represent the molecular mass of the glycoprotein polypeptide fraction (*right y axis*) as determined by the three-detector method (26).

To determine the oligomeric states of mouse decorin and its dimer interface mutants, we first used size exclusion chromatography with multiangle light scattering (SEC-MALS). Wild-type mouse decorin injected at 3 mg/ml concentration (83 μm) eluted in an asymmetric peak with a pronounced tail (Fig. 2). The molecular mass of the protein without modifications was determined to be 64.4 kDa (Table 1). This value is much closer to the calculated mass of a dimer (72.4 kDa) than that of a monomer (36.2 kDa). The dimer appears to dissociate quite readily, however, giving rise to an asymmetric peak and an average mass that is slightly lower than that of a dimer. The molecular mass of the glycoprotein (*i.e.* protein plus carbohydrate modifications) was determined to be 83.5 kDa (Table 1), which is in excellent agreement with the reported mass of 84.6 kDa for dimeric bovine decorin core glycoprotein (17). The elution profiles and molecular masses of the Y51A/R52A/Q54A and R151E mutants resembled those of the wild-type protein (Fig. 2 and Table 1), indicating that these mutations had not disrupted the mouse decorin dimer. In contrast, the Q61N and Y130N mutants eluted later than wild-type decorin and displayed symmetric peak profiles with molecular masses closely matching those of a monomer (Fig. 2 and Table 1). For the Q61N mutant, the disruption of the dimer could be attributed unequivocally to an engineered glycan as there is clear evidence for an additional modification in SDS-PAGE (Fig. 1*B*). For the Y130N mutant, the presence of a disruptive glycan could only be inferred from the observation that this mutant is monomeric. It is possible, although unlikely, that introduction of an unmodified asparagine at position 130 disrupts the decorin dimer. Because our objective was to obtain monomeric decorin mutants, we did not further investigate the presumed modification in the Y130N mutant.

**TABLE 1:** **Molecular masses of mouse decorin and its mutants as determined by SEC-MALS** The relative errors of the experimentally determined masses are <5%.

Protein	Calculated molecular mass	*N*-Linked glycosylation sites	Peak elution volume	Experimental mass of glycoprotein*^a^*	Experimental mass of polypeptide fraction*^b^*
	*kDa*		*ml*	*kDa*	*kDa*
WT	36.2	4	14.5	83.5	64.4
Y51A/R52A/Q54A	36.2	4	14.1	85.3	65.3
Q61N	36.2	5	15.5	52.1	37.6
Y130N	36.2	5	15.1	51.5	36.7
R151E	36.2	4	14.1	88.4	68.4

*^a^* Derived from the refractive index and light scattering signals.

*^b^* Derived from the absorbance, refractive index, and light scattering signals (26).

**FIGURE 3.**
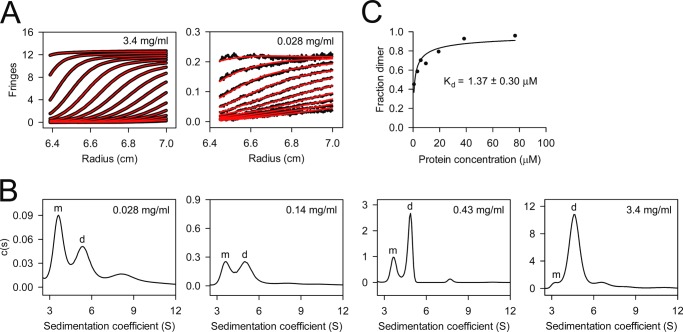
**Sedimentation velocity analysis of wild-type mouse decorin.** Seven concentrations from 0.028 to 3.4 mg/ml were analyzed in PBS at a rotor speed of 40,000 rpm. *A*, scan boundaries (*black*) and their fits (*red*) at the highest and lowest protein concentration. Only every third (3.4 mg/ml) or fifth (0.028 mg/ml) scan is shown for clarity. *B*, four continuous size distributions obtained from fitting the scan boundaries with the Lamm equation. The peaks assigned to monomeric (*m*) and dimeric (*d*) decorin are labeled. *C*, determination of the dimer dissociation constant. The dimer fractions were obtained by integration of the monomer and dimer peaks in the *c*(*s*) distributions. The *solid line* is a non-linear least square fit of the data by the equation describing a monomer-dimer equilibrium (see “Experimental Procedures”).

**FIGURE 4.**
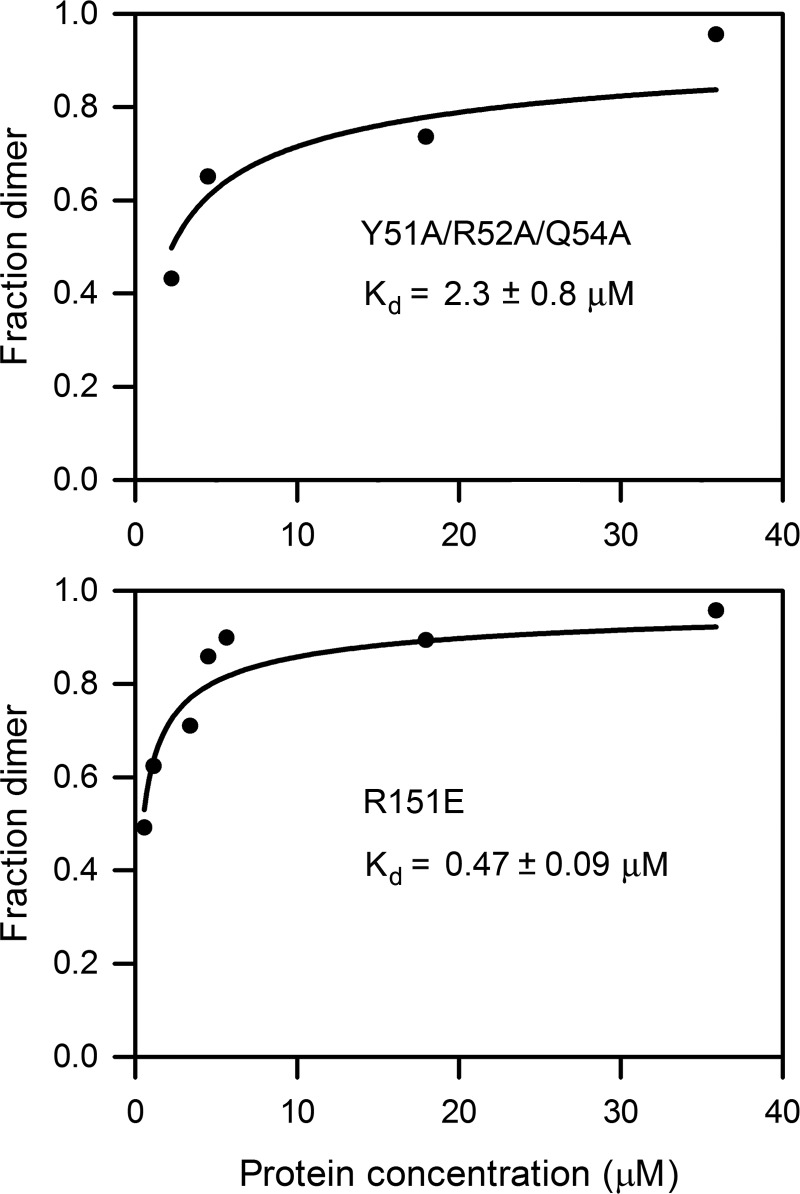
**Determination of the dimer dissociation constants of decorin mutants Y51A/R52A/Q54A and R151E by sedimentation velocity analysis.** The dimer fractions were obtained by integration of the monomer and dimer peaks in the *c*(*s*) distributions. The *solid lines* are non-linear least square fits of the data by the equation describing a monomer-dimer equilibrium (see “Experimental Procedures”).

The SEC-MALS experiment suggested that wild-type mouse decorin might exist in a concentration-dependent monomer-dimer equilibrium. To investigate the monomer-dimer equilibrium quantitatively, we used analytical ultracentrifugation. Using atomic models based on the crystal structure of bovine decorin (14) with appropriate carbohydrate modifications, we calculated *s*_20,*w*_^0^ values of 3.0 and 4.7 S for monomeric and dimeric mouse decorin (for details, see “Experimental Procedures”). We collected sedimentation velocity data at seven concentrations of wild-type decorin ranging from 0.028 to 3.4 mg/ml (0.77–94 μm) (Fig. 3*A*). The *c*(*s*) distributions derived from these data are characterized by two peaks, one at 3.6 S and one at 4.7–5.3 S, the relative proportions of which varied with the protein concentration (Fig. 3*B*). These peaks were interpreted to correspond to monomers and dimers, respectively, and their relative areas were used to derive a dissociation constant of 1.37 ± 0.30 μm for the mouse decorin dimer (Fig. 3*C*). Analogous experiments with the Y51A/R52A/Q54A and R151E mutants yielded comparable dimer dissociation constants of 2.3 ± 0.8 and 0.47 ± 0.09 μm, respectively (Fig. 4).

##### Stability of Wild-type and Mutant Decorin

**FIGURE 5.**
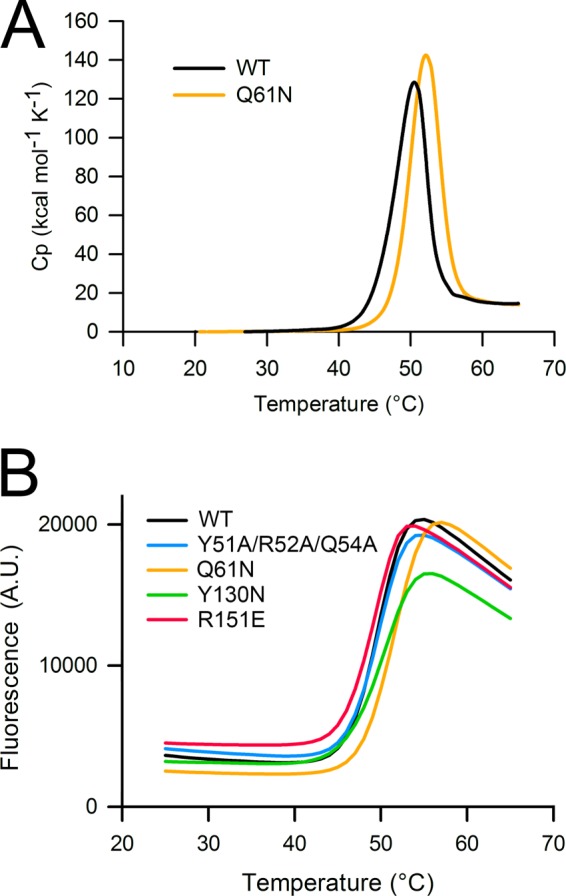
**Thermal stabilities of WT and mutant mouse decorin.** *A*, unfolding transitions obtained by differential scanning calorimetry at a protein concentration of 3 mg/ml in PBS. The melting temperatures derived from the peak maxima are 50 (WT) and 52 °C (Q61N), respectively. *B*, unfolding transitions obtained by differential scanning fluorimetry at a protein concentration of 0.181 mg/ml in PBS. The melting temperatures derived from the inflection points of the curves are 49 (R151E), 50 (WT and Y51A/R52A/Q54A), and 51 °C (Q61N and Y130N), respectively. Shown is a representative of two independent experiments carried out in triplicate. *A.U.*, arbitrary units.

The experiments described so far show that wild-type mouse decorin exists in a monomer-dimer equilibrium and that mutants can be obtained that behave as pure monomers in SEC-MALS. To quantify the thermal stabilities of selected proteins, we used differential scanning calorimetry. Wild-type mouse decorin and the monomeric Q61N mutant unfolded in single transitions with melting temperatures of 50 and 52 °C, respectively (Fig. 5*A*), which compare with a reported melting temperature of 46 °C for bovine decorin (18). Unfolding was only partially reversible as the signals on a second up-scan were reduced by ∼90% (data not shown). To extend the analysis to the remaining mutants, we used differential scanning fluorimetry, which monitors thermal unfolding using a hydrophobic dye and requires only small amounts of protein (31). The unfolding curves of all decorin proteins were very similar with inflection points ranging from 49 to 51 °C (Fig. 5*B*). As in the differential scanning calorimetry experiment, the monomeric mutants with engineered glycosylation sites (Q16N and Y130N) were marginally more stable than wild-type decorin. A positive effect of glycans on protein stability is well documented and frequently exploited in the pharmaceutical industry (32).

##### Collagen Binding by Wild-type and Mutant Decorin

**FIGURE 6.**
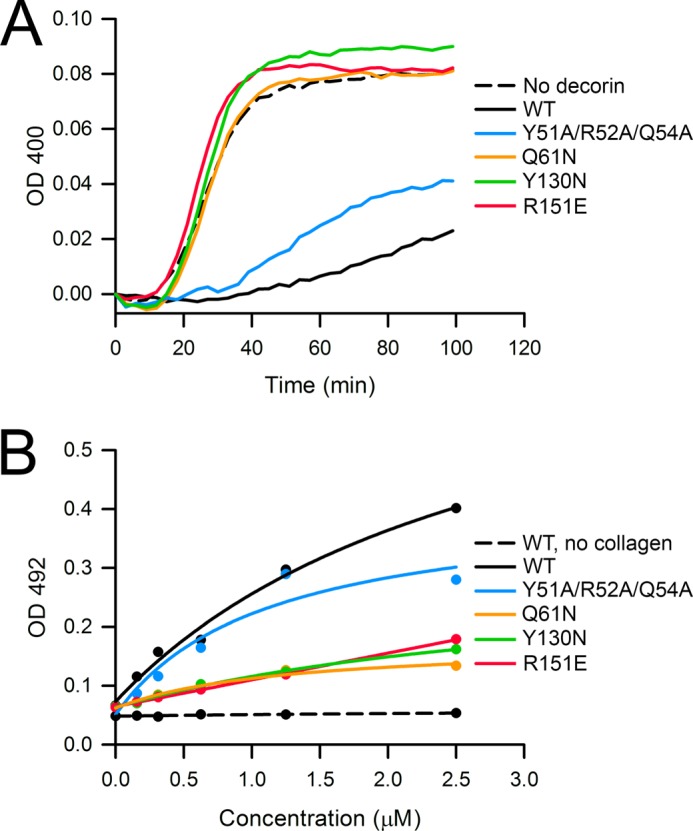
**Collagen binding by WT and mutant mouse decorin.** *A*, inhibition of collagen fibrillogenesis by WT and mutant mouse decorin. Type I collagen (32 μg/ml) was incubated at pH 7.8 and 37 °C, and the turbidity arising from fibril formation was recorded as absorbance at 400 nm. The decorin proteins were added at a concentration of 50 μg/ml. Shown is a representative of three independent experiments. *B*, collagen binding by WT and mutant mouse decorin. Type I collagen was immobilized on microtiter plates and incubated with varying amounts of decorin proteins. Bound decorin proteins were detected as absorbance at 492 nm using an antibody-linked color reaction. The *solid lines* are fits of the data by an equation describing single site binding. Shown is a representative of three independent experiments carried out in duplicate.

An important aim of the present study was to resolve the controversy whether decorin binds to collagen as a monomer or as a dimer (14, 19, 24). We first assessed collagen binding indirectly by measuring the inhibition of collagen fibrillogenesis, which is the classic assay for decorin activity (9). Wild-type mouse decorin robustly delayed fibrillogenesis of type I collagen (Fig. 6*A*) as reported previously for bovine and human decorin (10, 13, 14, 19, 22). Of the four dimer interface mutants, only the Y51A/R52A/Q54 mutant delayed fibrillogenesis similarly to wild-type protein. The Q61N, Y130N, and R151E mutants were completely inactive (Fig. 6*A*). We also attempted to measure collagen binding directly using a solid-phase assay with immobilized type I collagen (22, 33) but were frustrated by high and variable levels of nonspecific binding regardless of the blocking agent (albumin and casein) or detection method used (anti-mouse decorin antibody, anti-His tag antibody, biotinylation, and detection by avidin). Despite these problems, we consistently observed stronger collagen binding by wild-type mouse decorin and the Y51A/R52A/Q54A mutant than by any of the single point mutants (Fig. 6*B*). These observations corroborate the findings obtained with the more robust fibrillogenesis assay and indicate that decorin residues 61, 130, and 151 are important for collagen binding.

## DISCUSSION

Decorin and the related SLRPs biglycan, lumican, and fibromodulin play a major role in regulating collagen fibril formation in the extracellular matrix (2, 3), but how they bind to collagen has been unclear. A sterically plausible binding mode involves one or several collagen triple helices interacting with the concave face of the curved SLRP molecule (23, 24), which also happens to be the most highly conserved surface region (14). However, in crystal structures of decorin and biglycan, the concave face forms the interface of a seemingly tight dimer (14, 18), leading to controversy about the physiological relevance of the dimers (16–19). Here, we have resolved this controversy by showing that dimerization is reversible and that the concave face of decorin is involved alternatively in dimerization or collagen binding (Table 2).

**TABLE 2:** **Summary of results obtained with mouse decorin and its mutants**

Protein	Location of mutated residue(s)	Oligomeric state*^a^*	Collagen binding*^b^*
WT		Monomer-dimer equilibrium	Yes
Y51A/R52A/Q54A	N-terminal cap	Monomer-dimer equilibrium	Yes
Q61N	LRR1	Monomer	No
Y130N	LRR4	Monomer	No
R151E	LRR5	Monomer-dimer equilibrium	No

*^a^* Determined by SEC-MALS (Fig. 2) and analytical ultracentrifugation (Figs. 3 and 4).

*^b^* Determined by inhibition of collagen fibrillogenesis and solid-phase binding (Fig. 6).

Using analytical ultracentrifugation, we determined a dissociation constant of 1.37 μm for the mouse decorin dimer. A similar study of biglycan dimerization determined a dissociation constant of 4.5 μm (calculated from a free energy of association of −7.3 kcal/mol) (15). Thus, at the high concentrations typically used in solution scattering (17, 18) and crystallization experiments (14, 18), decorin and biglycan are dimers, but at plausible physiological concentrations, dimers will dissociate into monomers. In unfolding experiments with decorin and biglycan, denaturation coincides with a transition from (folded) dimer to (unfolded) monomer (17, 18). This finding has been interpreted as evidence that folded monomers cannot exist (18). By engineering glycosylation sites into the dimer interface, we have created decorin mutants (Q61N and Y130N) that remain monomeric at high concentration. The thermal stability of these mutants slightly exceeded that of wild-type decorin, likely due to a commonly observed stabilizing effect of engineered glycans (32). Thus, dimerization clearly is not essential to stabilize the decorin fold. Proteins that are structurally related to decorin and are stable monomers, such as Nogo receptor (34, 35) or LRR domain 3 of Slit (36), exist. Other proteins that dimerize similarly to decorin, such as LRR domain 4 of Slit (37) or AMIGO-1 (38), also exist. In contrast to our findings with decorin, mutation of interface residues in AMIGO-1 affected protein folding and secretion (38). Thus, the possibility remains that some SLRPs do not dissociate into stable monomers, but we believe that this is no longer a tenable scenario for decorin.

Engineering glycosylation sites into LRR1 (Q61N mutant) and LRR4 (Y130N mutant) or reversing the charge of a key residue in LRR5 (R151E mutant) abolished the ability of decorin to inhibit collagen fibrillogenesis and reduced collagen binding in a solid-phase binding assay. In contrast, a drastic triple mutation in the N-terminal cap region (Y51A/R52A/Q54A) had only a modest effect on collagen binding. Previous mutagenesis studies additionally implicate LRR6 (21) and LRR7 (22) in collagen binding. Thus, the picture that is emerging is that a large part of the concave surface of monomeric decorin may be involved in collagen binding. In agreement with this view, a recent modeling study using the experimentally derived structure of fibrillar type I collagen (39, 40) concluded that the decorin monomer could interact with up to six triple helices at the fibril surface (24).

An alternative interpretation of our results is that the monomeric decorin mutants Q61N and Y130N are inactive because collagen binding requires an intact decorin dimer (14). If this were the case, the R151E mutant, which dimerizes similarly to wild-type decorin, would be expected to bind collagen, but this is not the case. Using Equation 1 and the experimentally determined dimer dissociation constants, we estimate that 50% of wild-type decorin and 34% of the R151E mutant are available as monomers in the fibrillogenesis assay. This modest difference cannot explain the complete loss of collagen binding resulting from the R151E mutation. The simplest explanation is that Arg-151 (and the concave face as a whole) is directly involved in the binding of monomeric decorin to collagen fibrils. The ultimate proof for this model would require a mutant that is monomeric and binds collagen like wild-type decorin. Such mutants may well be elusive given that the dimer interface is formed precisely by the region most likely responsible for collagen binding.

## Footnotes

2 The abbreviations used are:

SLRP: small leucine-rich proteoglycan
Gal: galactose
GlcNAc: *N*-acetylglucosamine
LRR: leucine-rich repeat
Man: mannose
NeuNAc: *N*-acetylneuraminic acid
SEC-MALS: size exclusion chromatography with multiangle light scattering.
